# A Novel System for Real-Time, In Situ Monitoring of CO_2_ Sequestration in Photoautotrophic Biofilms

**DOI:** 10.3390/microorganisms8081163

**Published:** 2020-07-31

**Authors:** Patrick Ronan, Otini Kroukamp, Steven N. Liss, Gideon Wolfaardt

**Affiliations:** 1 Department of Chemistry and Biology, Ryerson University, 350 Victoria St., Toronto, ON M5B 2K3, Canada; pronan@ryerson.ca (P.R.); mkroukam@ryerson.ca (O.K.); steven.liss@ryerson.ca (S.N.L.); 2 Department of Microbiology, Stellenbosch University, Private Bag X1, Matieland 7602, South Africa

**Keywords:** biofilms, CO_2_ sequestration, microalgae, photosynthesis, real-time monitoring

## Abstract

Climate change brought about by anthropogenic CO_2_ emissions has created a critical need for effective CO_2_ management solutions. Microalgae are well suited to contribute to efforts aimed at addressing this challenge, given their ability to rapidly sequester CO_2_ coupled with the commercial value of their biomass. Recently, microalgal biofilms have garnered significant attention over the more conventional suspended algal growth systems, since they allow for easier and cheaper biomass harvesting, among other key benefits. However, the path to cost-effectiveness and scaling up is hindered by a need for new tools and methodologies which can help evaluate, and in turn optimize, algal biofilm growth. Presented here is a novel system which facilitates the real-time in situ monitoring of algal biofilm CO_2_ sequestration. Utilizing a CO_2_-permeable membrane and a tube-within-a-tube design, the CO_2_ sequestration monitoring system (CSMS) was able to reliably detect slight changes in algal biofilm CO_2_ uptake brought about by light–dark cycling, light intensity shifts, and varying amounts of phototrophic biomass. This work presents an approach to advance our understanding of carbon flux in algal biofilms, and a base for potentially useful innovations to optimize, and eventually realize, algae biofilm-based CO_2_ sequestration.

## 1. Introduction

Over the past two centuries, human activity has had a pronounced effect on the natural environment [1]. Anthropogenic CO_2_ emissions have led to a range of detrimental environmental impacts, including the acidification of ocean waters, rising sea levels, and increasing global temperatures [2,3]. Accordingly, climate change mitigation and CO_2_ management have become a top priority globally, which is increasingly reflected in our political, social, and economic realms.

Engineered bio-sequestration systems which exploit the natural CO_2_-capturing ability of photosynthetic plants and algae potentially represent a viable and valuable compliment to current CO_2_ management strategies [4]. Microalgae are particularly well suited for this, given their superior growth and CO_2_-fixation rates compared to terrestrial plants [5], as well as the commercial value of their biomass. For example, microalgal biomass can be used as animal feed and fertilizer with minimal downstream processing required [6]. It is also a source of numerous valuable compounds such as pigments, cosmetic products, and nutraceuticals like omega-3 fatty acids [5,7,8], and the lipid-rich nature of algal cells makes them an attractive biodiesel feedstock [9,10,11].

The commercial value of microalgal biomass enables a direct link between CO_2_ sequestration and the generation of value-added products which can be used to subsidize the overall process [7]. Despite the benefits of such an approach, there is a need for further research focusing on developing new tools, growth systems, and methodologies to make this an economically viable, large-scale option [3,12]. Notably, the ability to track and measure the culture’s CO_2_ uptake rate and efficiency in real time is crucial in order to allow for process monitoring, assessment, and optimization.

To date, studies exploring microalgal CO_2_ sequestration and biomass production have focused predominantly on suspended cultures [13,14]. In nature, however, microalgae, like most microorganisms, commonly grow as aggregates in suspension, or attached to solid surfaces as biofilms. The cells within these aggregates are encased in a matrix of extracellular polymeric substances (EPSs) and exhibit markedly different physiological and metabolic characteristics compared to single planktonic cells of the same species [15]. From an algae cultivation perspective, biofilm growth systems offer several key advantages over the more conventional suspended growth systems. For example, the time, energy, and financial costs associated with biomass harvesting and dewatering are lessened significantly [14]. Immobilization of the cells in biofilm growth systems creates the potential for more efficient use of light through the positioning of concentrated biomass for optimum light harvesting [16,17].

Conventionally, CO_2_ uptake is assessed by weighing the algal biomass produced and, using an approximate algal cell carbon content of 40–50%, stoichiometrically calculating the amount of carbon sequestered [18,19]. However, since this is a destructive measurement, it provides little insight about a biofilm’s performance during active growth. Furthermore, the fact that many algal strains are also able to metabolize organic carbon through mixotrophic growth can make such calculations problematic [20]. Another means of quantifying algal CO_2_ sequestration is by measuring effluent pH. As dissolved CO_2_ is taken up and fixed, the resulting effluent pH rise can be measured and used as an indirect indicator of changes in CO_2_ concentration. However, since other factors such as nitrogen assimilation can also influence effluent pH, CO_2_ quantification on this basis alone may be inaccurate [21]. Lastly, CO_2_ electrodes exist which can be used to monitor dissolved CO_2_ concentrations. However, such probes can be prohibitively expensive, become fouled, and are often not adequately sterilizable [21]. Therefore, a system which can accomplish non-destructive monitoring of CO_2_ sequestration by photoautotrophic biofilms in real time is strongly desirable.

Presented here is a novel system for the real-time in situ monitoring of biofilm bio-sequestration. Building upon the CO_2_ evolution measurement system (CEMS) first described by Kroukamp and Wolfaardt [22], the present CO_2_ sequestration monitoring system (CSMS) was used to quantify the CO_2_ uptake rate of photoautotrophic biofilms under various conditions. The data presented demonstrates the ability of the CSMS to detect and monitor CO_2_ flux during (i). light–dark cycling, (ii). long- and short-duration light intensity shifts, and (iii). changing amounts of photoautotrophic biomass in the system. This work presents an approach to advance our understanding of carbon flux in algal biofilms, carbon exchange between autotrophic and heterotrophic biofilms, as well as a base for potentially useful innovations to optimize, and eventually realize, algae biofilm-based CO_2_ sequestration.

## 2. Materials and Methods

### 2.1. System Configuration

**Figure 1:**
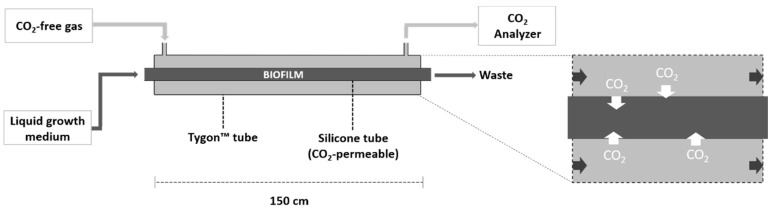
Schematic of the biofilm reactor (BR) module. Liquid medium is pumped into the silicone tube where inoculation and subsequent biofilm growth occur. The annular space created by the Tygon™ tube is used for gas channeling (dark arrows). CO_2_ molecules can readily pass between the dry, gaseous environment in the annular space and the aqueous environment inside the silicone tube (white arrows). The CO_2_ analyzer downstream continuously monitors the CO_2_ concentration in the gas stream.

The CSMS used in this study comprises two distinct biofilm reactor (BR) modules. The first, referred to as BR_prod_ (CO_2_ *producer*), is used to grow a heterotrophic bacterial biofilm, which serves as a biotic source of concentrated CO_2_. The second, BR_cons_ (CO_2_ *consumer*), is used to grow the photoautotrophic biofilm of interest. Each BR consists of a tube-within-a-tube design (Figure 1).

Biofilms were grown inside a CO_2_-permeable silicone tube (1.57 mm ID, 2.41 mm OD, 0.41 mm wall thickness; VWR International, Mississauga, ON, Canada) 150 cm in length, with a volume of 2.9 mL and approximately 74 cm^2^ of colonizable surface area. Housing this tube was a larger diameter CO_2_-impermeable Tygon™ tube also 150 cm in length (4.76 mm ID, 7.94 mm OD, 1.58 mm wall thickness, E-3603 formulation; VWR International, Mississauga, ON, Canada). The total volume of this tube, accounting for the volume occupied by the inner silicone tube, was 19.85 mL (Table 1). Protruding near the influent and effluent end of the outer Tygon™ tube was smaller diameter Tygon™ tubing (1.59 mm ID, 3.18 mm OD, 0.79 mm wall thickness, E-3603 formulation; VWR International, Mississauga, ON, Canada). Sealant was used to ensure the connections were airtight.

**Figure 2:**
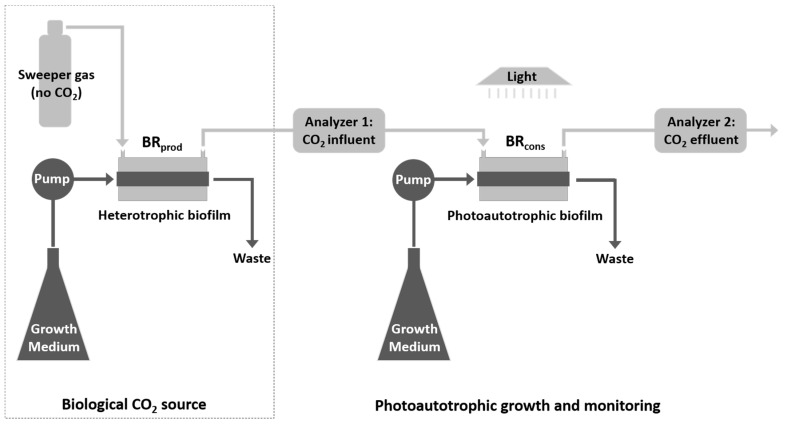
Configuration of the CSMS. CO_2_ produced by the heterotrophic biofilm in BR_prod_ (CO_2_ *producer*) enters the annular space where it is carried by the sweeper gas to BR_cons_ (CO_2_ *consumer*) via Analyzer 1. As the photoautotrophic biofilm inside BR_cons_ develops, its rate of CO_2_ uptake increases, causing a subsequent decrease in the amount of gaseous CO_2_ reaching Analyzer 2. The difference in CO_2_ concentration logged by Analyzer 1 and 2 provides a real-time measure of the biofilm’s CO_2_ uptake.

Liquid growth medium pumped through the inner silicone tube provided nutrients to the biofilm. The silicone membrane has a high CO_2_ permeability (2013.2 Barrer), which allowed CO_2_ molecules to readily pass between the aqueous environment inside this tube, and the dry, gaseous environment in the annular space. BR_prod_ and BR_cons_ were separately supplied with sterile growth medium to grow heterotrophic bacterial, and photoautotrophic biofilms, respectively. In contrast, the gas flow in the annular space was continuous through both BRs (Figure 2), allowing the heterotrophic biofilm in BR_prod_ to serve as a biotic source of CO_2_ to the photoautotrophic biofilm growing inside BR_cons_.

### 2.2. Gas Channeling and Monitoring

A sweeper gas of CO_2_-free air (TOC grade, 24001980; Linde Canada Limited, Concord, ON, Canada) was channeled into the annular space of the system at a constant flow rate of 2.5 mL/min using a mass flow controller (GFC17 mass flow controller; Aalborg Instruments & Controls, Inc., Orangeburg, NY, USA). This carried CO_2_ produced by the heterotrophic biofilm in BR_prod_ to BR_cons_ via a non-dispersive infrared CO_2_ analyzer (Analyzer 1) (LI-820; LI-COR Biosciences, Lincoln, NE, USA). The analyzer has a precision in the range of ~1 ppm and was used to measure and log the CO_2_ concentration at one-minute intervals.

As the photoautotrophic biofilm inside BR_cons_ developed, its rate of CO_2_ uptake increased, as reflected by the consequent decrease in the amount of gaseous CO_2_ reaching Analyzer 2, positioned downstream of BR_cons_ (Figure 2). The difference in CO_2_ concentration logged by Analyzers 1 and 2 (BR_cons_ CO_2_ influent and effluent, respectively) thus provided a real-time measure of CO_2_ uptake by the photoautotrophic biofilm. By default, the analyzers record CO_2_ concentration in parts per million (ppm). However, given a gas flow rate of 2.5 mL/min and the fact that the temperature and pressure inside the CO_2_ analyzers are also recorded (typically 50 °C and 100 kPa, respectively), the ideal gas law can be applied to convert these raw ppm values to rates of CO_2_ uptake, provided in units of µmol/h.

### 2.3. Light Exposure and Intensity

The photoautotrophic biofilm in BR_cons_ was illuminated with a fluorescent plant-growth light (JSV2 Jump Start T5 Grow Light System; Hydrofarm Inc., Petaluma, CA, USA). The bulb provided simulated sunlight with a 6400 K color temperature. This lighting system affords the ability to raise and lower the light as desired. Unless otherwise stated, illumination was provided 5 cm above the biofilm, resulting in a photosynthetic photon flux density (PPFD) of approximately 141.79 µmol/m^2^/s.

### 2.4. Growth Media

The heterotrophic biofilm in BR_prod_ was fed a tryptic soy broth at a flow rate of 0.25 mL/min using a Watson Marlow 205S peristaltic pump. This medium was prepared as a 0.6 g/L solution, which is a 2% concentration relative to the manufacturer’s directions for typical batch experiments. The resulting composition was 0.34 g/L casein peptone, 0.06 g/L soya peptone, 0.1 g/L sodium chloride, 0.05 g/L dipotassium hydrogen phosphate, and 0.05 g/L glucose. Media were prepared in distilled water and autoclaved at 121 °C for 20 min prior to use.

The photoautotrophic biofilm in BR_cons_ was fed a modified Bold’s Basal Medium (M-BBM) (also at 0.25 mL/min) with a composition of 0.22 g/L (NH_4_)_2_SO_4_, 0.025 g/L NaCl, 0.025 g/L CaCl_2_ · 2H_2_O, 0.075 g/L MgSO_4_ · 7H_2_O, 0.175 g/L KH_2_PO_4_, 0.075 g/L K_2_HPO_4,_ 8.34 mg/L FeSO_4_. It should be noted that no carbon was provided in the liquid medium. All medium components were maintained in filter-sterilized 100× stock solutions, which were combined and diluted in distilled water and autoclave sterilized at 121 °C for 20 min prior to use. The CSMS represents a plug flow reactor, with the BR module having a retention time of 11.6 min and a dilution rate of 5.17 h^−1^. This greatly exceeds the maximum specific growth rate values of microalgal and bacterial strains [23,24,25], which ensures that non-biofilm-bound cells are readily washed out the system.

### 2.5. Test Cultures and System Inoculation

BR_prod_ and BR_cons_ were inoculated with a heterotrophic and photoautotrophic culture, respectively. The heterotrophic culture used was *Pseudomonas aeruginosa* (PA01) [26], previously labelled with GFP via a mini-Tn7 transposon system [27]. For each CSMS experiment, PA01 was inoculated as an overnight culture, prepared by transferring one colony from a tryptic soy agar plate into 10 mL of 0.6 g/L tryptic soy broth and incubated overnight on a rotary shaker at 30 °C.

BR_cons_ was the “test module” in which the photoautotrophic biofilm of interest was grown and studied. The culture used was initially enriched from an aerated wastewater lagoon in Dundalk, Ontario, Canada. A 5 mL sample was inoculated into 145 mL of M-BBM and illuminated continuously with the fluorescent plant growth light. Filtered ambient air was bubbled into the culture to provide mixing and dissolved CO_2_. When the culture was sufficiently dense (qualitatively green and turbid), a 10 mL aliquot was transferred to 90 mL of fresh medium and placed under the same conditions. This transferring procedure was repeated biweekly, ensuring that the photoautotrophic culture inoculated into BR_cons_ was not more than 14 days old.

Sterile syringes were used to inoculate reactors by injecting 3 mL of the appropriate culture into the respective BRs through the silicone tubing just upstream of the reactor. A 22-gauge hypodermic syringe needle was used to pierce the tubing, and a dollop of silicone glue was used to seal this puncture as the needle was removed. Liquid medium flow was paused during inoculation and for a period of one hour following this to allow for some cell adherence.

The BRs were inoculated concurrently, with the exception of the first experiment. In that case, BR_cons_ was inoculated with the photoautotrophic culture approximately 24 h after the inoculation of PA01 into BR_prod_, once the CO_2_ output of the heterotrophic biofilm had plateaued. This allowed for the assessment of CO_2_ uptake during early stage photoautotrophic biofilm development, which was not possible when the BRs were inoculated concurrently. In subsequent experiments, the CO_2_ uptake data is presented for mature photoautotrophic biofilms of at least 20 h old.

### 2.6. Assessing CO_2_ Uptake during Photoautotrophic Biofilm Development

A photoautotrophic culture was inoculated into BR_cons_ approximately 24 h after BR_prod_ was inoculated with PA01. This ensured that a relatively steady concentration of CO_2_ was achieved at the time of BR_cons_ inoculation and allowed for the visualization of CO_2_ uptake during photoautotrophic biofilm development. Taking the difference in CO_2_ concentration measured by Analyzer 1 and 2 at each time point gave a raw concentration in parts per million, which, using the ideal gas law, was converted to a rate of CO_2_ uptake and plotted against time.

To confirm the presence and photosynthetic activity of the photoautotrophic biofilm, a procedure similar to the light–dark shift method described by Revsbech et al. [28] was performed, in which a steady state biofilm in BR_cons_ was covered with an opaque sheet for approximately four hours and the resulting shift in CO_2_ flux was recorded.

A series of abiotic control experiments were also conducted to confirm that any difference in BR_cons_ influent and effluent CO_2_ concentration in CSMS experiments was in fact due to the photoautotrophic activity of the biofilm, and not leakage or passive diffusion out of the system. This was achieved by channeling concentrated CO_2_ (1800 ppm, 24081376, Linde Canada Limited, Concord, ON, Canada) through a sterile, un-inoculated CSMS at two different flow rates (2.5 mL/min and 20 mL/min) for a period of 0.25 h.

### 2.7. Light Manipulations

#### 2.7.1. CO_2_ Uptake during Light–Dark Cycling

A series of tests were performed to demonstrate the ability of the CSMS to detect real-time changes in CO_2_ uptake by the photoautotrophic biofilm, brought about by manipulations of light illumination. First, the system was operated on a diurnal cycle (12 h light, 12 h dark), with the photoautotrophic biofilm’s CO_2_ uptake rate assessed and measured as µmol/h. The system was covered in such a way as to ensure that no light could reach the biofilm during the dark periods, and that only light from the fluorescent growth light could illuminate the biofilm during light periods. The light/dark switching was accomplished using a programmable plug-in timer. This experiment lasted for 120 h, representing a total of five light–dark cycles.

#### 2.7.2. CO_2_ Uptake during Changes in Light Intensity

To investigate the CSMS’s ability to detect changes in CO_2_ uptake brought about by changes in light intensity, the biofilm was first grown under constant illumination with the light source suspended 45 cm above the biofilm. This light intensity resulted in a relatively low PPFD of 25.57 µmol/m^2^/s. After 2 days of growth, the light source was lowered to 5 cm above the biofilm for a period of 20 h, resulting in an increased PPFD of approximately 141.79 µmol/m^2^/s. The light source was then returned to its original height for an additional 12 h.

A similar experiment was conducted in which the light intensity illuminating an actively growing, mature biofilm was manipulated on a significantly shorter time scale. A mature photoautotrophic biofilm was initially illuminated from a height of 5 cm. The light was then raised to 45 cm, corresponding to a nearly ten-fold decrease in PPFD, for approximately 90 min. Following this, the biofilm was kept in complete darkness for 90 min, before being illuminated again for 90 min from 45 and 5 cm, respectively.

### 2.8. Variation in Biofilm Biomass

#### 2.8.1. Assessing CO_2_ Uptake with Supplementary Photoautotrophic Biofilm

To demonstrate the modular nature of the CSMS, a third BR (BR_cons2_) was added to the system immediately downstream of Analyzer 2. The same photoautotrophic culture that was inoculated into BR_cons_ was also inoculated into BR_cons2_. BR_cons2_ received its own feed of fresh M-BBM. The gas stream in the system however was sequential, with the sweeper gas flowing from Analyzer 2, through the annular space of BR_cons2_ to a third CO_2_ analyzer (Analyzer 3).

#### 2.8.2. Assessing CO_2_ Uptake with Increased Photoautotrophic Biofilm Length

A similar experiment utilizing a third BR was conducted with sweeper gas flowing directly from BR_cons_ via Analyzer 2 to BR_cons2_ and finally Analyzer 3. However, this time, the medium flow was also setup in sequence, such that the effluent of BR_cons_ served as growth medium for BR_cons2_. Six mL of the photoautotrophic culture was injected into the silicone tubing just upstream of BR_cons_, which was enough to ensure that the inoculum reached the end of BR_cons2_. In this orientation, BR_cons_ and BR_cons2_ represented two halves of one biofilm approximately 300 cm in length, with the positioning of Analyzer 2 between BR_cons_ and BR_cons2_ allowing for the measurement of CO_2_ flux by each half of the biofilm separately.

## 3. Results and Discussion

### 3.1. Development of Algal Biofilms

**Figure 3:**
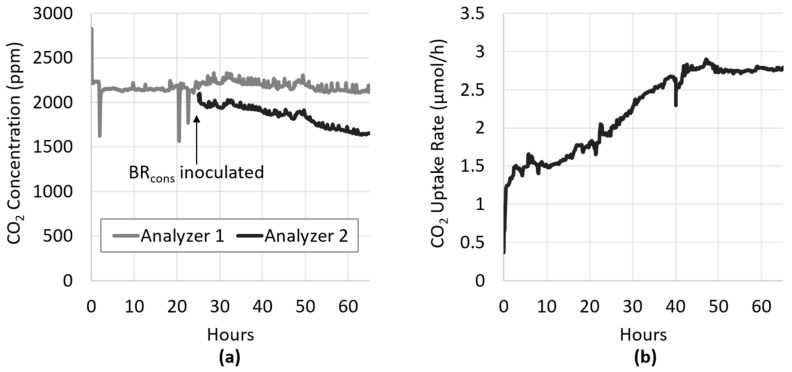
BR_cons_ was inoculated with the photoautotrophic culture approximately 24 h after inoculation of the heterotrophic culture into BR1. (**a**) Divergence between BR_cons_ influent and effluent CO_2_ concentration (Analyzer 1 and 2, respectively) was apparent within several hours of inoculation. (**b**) Using the ideal gas law, the difference between the influent and effluent concentration was converted to the rate of CO_2_ uptake, which resembled a typical microbial growth curve.

**Figure 4:**
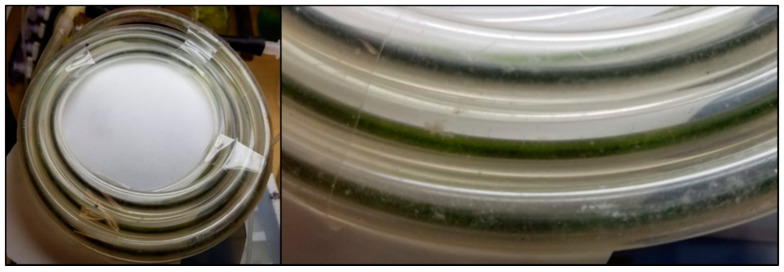
Image and close up of BR_cons_ colonized by a mature photoautotrophic biofilm. Green biomass is apparent within the inner silicone tube.

Algal biofilm growth became evident in BR_cons_ within several hours after inoculation. The inflow and outflow CO_2_ concentrations over the reactor began to diverge due to increased capture by the developing photosynthetic biofilm (Figure 3a). Conversion of concentration data into CO_2_ uptake rate resembles a typical microbial growth curve (Figure 3b), with a green biofilm visible (Figure 4) when the uptake rate levelled off at approximately 2.8 µmol/h (378 µmol/h/m^2^). 

In order to confirm that the divergence depicted in Figure 3a between Analyzer 1 (BR_cons_ gas influent) and Analyzer 2 (BR_cons_ gas effluent) was attributable to the activity of the photoautotrophic biofilm in BR_cons_, a procedure similar to the “light–dark shift method” [28] was performed. Briefly, this well-established method provides an assessment of photosynthetic activity by measuring the decrease in dissolved oxygen concentration that occurs when the specimen is placed in the dark after prolonged light exposure [28]. Originally described in the early 1980s, this method was developed to measure primary production in benthic sediments. Here, a similar procedure was used, whereby a photoautotrophic biofilm in BR_cons_ was placed in the dark and the change in CO_2_ flux within it was observed and recorded.

**Figure 5:**
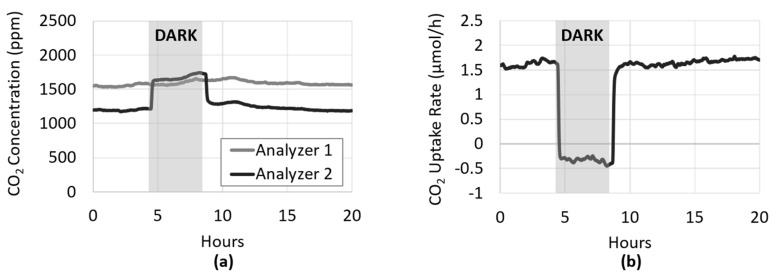
A steady state photoautotrophic biofilm was placed in the dark by covering BR_cons_ with an opaque sheet for approximately 4 h. Time zero refers to twenty hours post inoculation. (**a**) Within minutes, the CO_2_ concentration in the BR_cons_ gas effluent (Analyzer 2) increased rapidly and remained elevated for the duration of the dark period. After illumination resumed, the effluent concentration quickly returned to its pre-dark level. (**b**) When converted and plotted as the rate of CO_2_ uptake, the plot depicts a complete cessation of photosynthesis and CO_2_ fixation in the dark, demonstrating the presence and activity of the photoautotrophic biofilm in BR_cons_.

As shown in Figure 5a, the dark response was rapid, with the CO_2_ concentration in BR_cons_ effluent increasing sharply to the same level as the influent, and ultimately surpassing it in less than 10 min. This higher CO_2_ concentration was likely due to some baseline respiration by the algae that proceeded in the dark. When plotted as the rate of CO_2_ uptake (Figure 5b), the effect of darkness on the biofilm’s activity was clearly apparent, demonstrating a complete cessation of photosynthesis. This provided evidence that the divergence observed between BR_cons_ influent and effluent CO_2_ concentration was in fact due to CO_2_ fixation by the growing photoautotrophic biofilm. When the flow of photons to the biofilm is halted, the light-dependent photosynthetic reaction stops, and the ATP and NADPH needed to power the Calvin–Benson cycle and CO_2_ fixation is depleted, which happens rapidly as demonstrated by the data.

The rapid dark response observed in the CSMS coincides with previous results using the light–dark shift method, where dramatic changes in photosynthetic rate were detected in a matter of seconds after switching to dark conditions [29]. Given that the CSMS is a closed, continuous flow system, one can expect a small lag in measurement time related to the length of tubing linking BR_cons_ to Analyzer 2. This, however, can be mitigated by reducing this distance, such that the time needed for the gas to travel from the biofilm to the CO_2_ analyzer is minimized.

**Figure 6:**
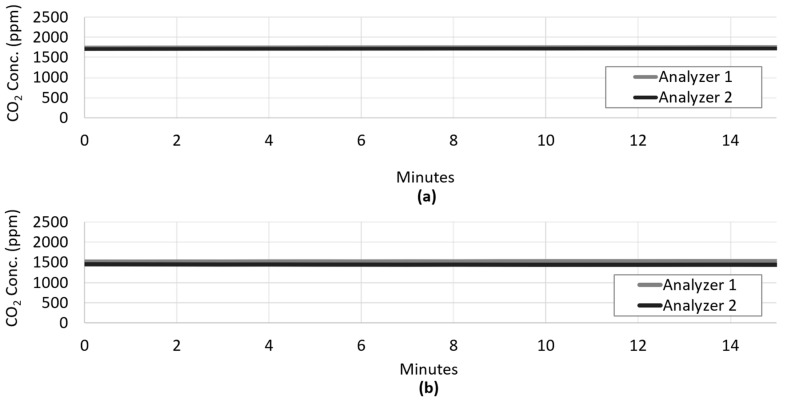
Abiotic control tests were performed in which concentrated CO_2_ was channeled through the uninoculated system for 0.25 h at a flow rate of 20 mL/min (**a**), and 2.5 mL/min (**b**). In both cases, the influent and effluent CO_2_ concentration remained virtually unchanged, demonstrating that without assimilation by the photoautotrophic biofilm, CO_2_ loss was minimal.

To further confirm that the divergence between influent and effluent CO_2_ concentration is due to the presence of the photoautotrophic biofilm, a set of abiotic control experiments were conducted. When concentrated CO_2_ was channeled through the uninoculated system at two flow rates, the influent and effluent CO_2_ concentration remained virtually unchanged, demonstrating that without assimilation by the photoautotrophic biofilm, CO_2_ loss was minimal (Figure 6).

The system described here may allow more efficient capture of CO_2_ than conventional bioreactor configurations, since its bulk supply is in the gas phase and therefore not limited to its solubility in water. Gas transport across the silicone wall was described in detail in [22] when the bulk solutions on the opposite sides are water and air, respectively, which was followed by experiments that showed high linearity (R^2^ = 0.999) between CO_2_ measured in the gas phase and various dissolved CO_2_ concentrations. In the present study, CO_2_ transport in the opposite direction was measured. Gas permeability of silicon has found applications such as gas exchange in blood [30] as well as membrane gills for submarines and underwater stations [31]. Here, we explored the potential utility of such permeability in microbial systems with biotechnological relevance.

### 3.2. Light Manipulation

**Figure 7:**
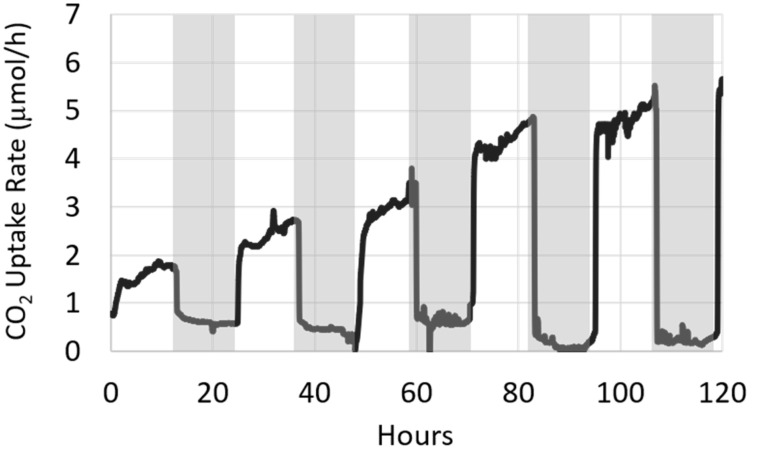
The photoautotrophic biofilm in BR_cons_ was illuminated on a cycle of alternating 12 h light and dark periods. On–off toggling of biofilm photosynthesis and CO_2_ uptake is readily apparent. Following each dark period, CO_2_ uptake rebounded to approximately the same level reached at the end of the previous light period. Time zero refers to twenty hours post inoculation.

Figure 7 depicts the CO_2_ uptake rate of the photoautotrophic biofilm grown under alternating 12 h light and dark periods, demonstrating the biofilm’s photosynthesis on–off toggling during light–dark cycling. The data shows a net increase in CO_2_ consumption during the light phases with increasing biofilm age. At the end of each dark period, the biofilm’s CO_2_ uptake rate rapidly rebounded to approximately the rate reached at the end of the previous light period, ultimately achieving a rate of approximately 5.5 µmol/h (743 µmol/h/m^2^) by the last light period. There was also a small, decreasing rate of CO_2_ uptake in each sequential dark phase. Although we do not expect CO_2_ consumption by direct biologically related reactions during periods of darkness, this may be the result of other biotic or abiotic processes (e.g., changes in pH) which would have affected the CO_2_-bicarbonate equilibrium.

Since the overarching industrial goal of bio-sequestration is to maximize the amount of CO_2_ captured, it may be tempting to provide the sequestering culture with continuous 24 h illumination. This would prevent the cessation of CO_2_ fixation which occurs in the dark and promote higher biomass accumulation rates. However, dark periods are important in allowing for the repair of photosynthetic machinery and proteins, which may benefit the culture’s long-term performance [5]. A key operational decision in bio-sequestration systems, therefore, is how to achieve maximum CO_2_ capture while maintaining the long-term health of the sequestering culture. Biofilm accumulation is not an infinite process, and ultimately slows down to a quasi-steady state as dictated by factors such as flow regimes and gas exchange/transport through the biofilm matrix, which will lead to a simultaneous lowering of CO_2_ uptake. By enabling real-time in situ monitoring of the biofilm’s CO_2_ uptake rates, the CSMS may offer an approach to manage reactor performance, such as timing of harvesting, while real-time CO_2_ uptake rates may be useful in setting an optimal light–dark cycling regimen.

Photosynthesis is driven by photon energy that excites electrons in the chloroplasts’ photosynthetic pigments. Through a cascade of redox reactions, this electron energy is transiently stored as ATP and NADPH, which in turn are used in the Calvin–Benson cycle to fix CO_2_ and generate glucose. Generally, a higher density of photons irradiating the cell will lead to a higher rate of CO_2_ fixation. That is, however, before an upper light intensity threshold is reached and photoinhibition occurs. This critical threshold is a product of numerous abiotic and biotic factors including light wavelength(s) and color temperature, growth system configuration and material properties, as well as culture cell density [5]. As such, it is extremely difficult to accurately predict the onset of photoinhibition of a culture in a given system without some degree of real-time monitoring of culture performance.

**Figure 8:**
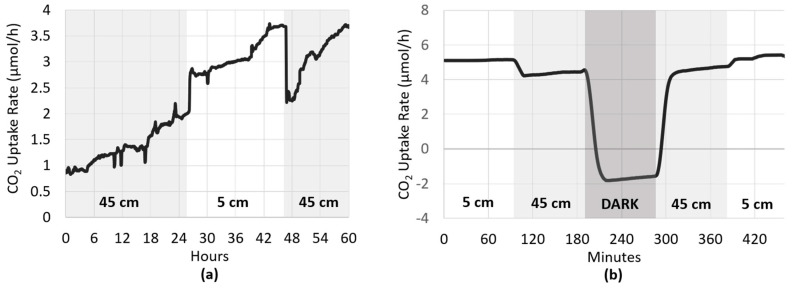
To assess whether the CSMS can detect changes in the biofilm’s CO_2_ uptake rate brought about by small fluctuations in light intensity, the height of light source illuminating BR_cons_ was adjusted on both long (**a**) and short (**b**) timescales. Heights of 5 and 45 cm refer to PPFDs of approximately µmol/m^2^/s and 25.57 µmol/m^2^/s, respectively.

The next series of tests was conducted to assess the ability to detect changes in the biofilm’s CO_2_ uptake rate brought about by fluctuations in light intensity. This was done on both long (Figure 8a) and short (Figure 8b) timescales. Figure 8a shows the CO_2_ uptake rate of a photoautotrophic biofilm growing in the system with the fluorescent growth light initially placed at a height of 45 cm above the biofilm, corresponding to a relatively low PPFD of 25.57 µmol/m^2^/s. During this period, the CO_2_ uptake rate gradually increased from approximately 0.8 to 2 µmol/h (108 to 270 µmol/h/m^2^). After 24 h, the light was lowered to 5 cm above the biofilm, resulting in an increased light intensity and PPFD of 141.79 µmol/m^2^/s. Within minutes, the biofilm’s CO_2_ uptake rate increased sharply by approximately 40%, before continuing to gradually increase at roughly the same trajectory as before the light intensity shift. After 20 h, the growth light was returned to a height of 45 cm, and the biofilm once again exhibited a near immediate response with its CO_2_ uptake rate rapidly falling by approximately 40% to 2.2 µmol/h (297 µmol/h/m^2^). Similarly, when the light intensity was adjusted on a much shorter time scale (Figure 8b), the same rapid responses were observed. After 100 min in the dark, it took only 20 min for the biofilm’s CO_2_ uptake rate to return to its pre-dark level.

Phototrophic biofilms typically consist of a photosynthetically active zone, which is closest to the light-source, and a photosynthetically inactive zone where cells are shaded by the cells of the active layer. With increasing light intensities, photon flux penetrates deeper into the biofilm and the thickness of the photosynthetically active layer increases [14]. Therefore, at higher light intensities, a larger proportion of the biofilm is photosynthetically active, and its gross CO_2_ uptake increases, as depicted in Figure 8. Collectively, these results coincide with the notion that increasing light intensity leads to a higher rate of CO_2_ fixation, and demonstrates the ability of the CSMS to detect real-time changes in CO_2_ uptake caused by small fluctuations in light intensity. This system therefore also has utility in its ability to monitor and alert when light intensities are above the critical threshold, which would otherwise cause photoinhibition and consequent diminished productivity.

### 3.3. Variation in Biofilm Biomass

**Figure 9:**
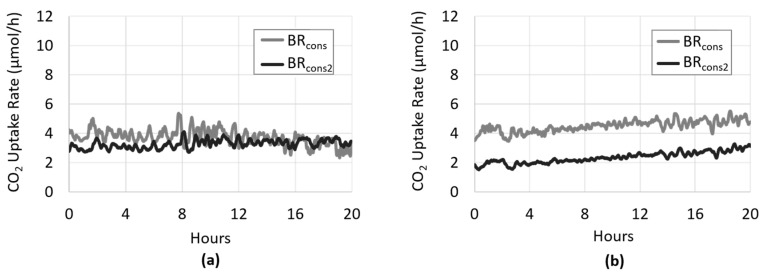
The CSMS was amended with an additional biofilm reactor (BR_cons2_) downstream of BR_cons_. The gas stream in the system was sequential, with the sweeper gas flowing from Analyzer 2, through the annular space of BR_cons2_ to a third CO_2_ analyzer (Analyzer 3). (**a**) Initially, BR_cons_ and BR_cons2_ were each inoculated with the photoautotrophic culture separately and received its own fresh feed of growth medium. In this configuration, the CO_2_ uptake rate within BR_cons_ and BR_cons2_ were approximately the same, meaning that the presence of a second photoautotrophic biofilm roughly doubled the system’s overall CO_2_-sequestering capacity. (**b**) Subsequently, the system was configured such that both medium and gas passed sequentially from BR_cons_ to BR_cons2_, producing a biofilm the length of two BRs. In this case, the CO_2_ uptake rate in the first half of the biofilm (BR_cons_) is higher than the second half of the biofilm (BR_cons2_), likely due to the depletion of some key nutrient(s) by the time the growth medium reached BR_cons2_.

Amongst the main objectives of bio-sequestration is to maximize the amount of CO_2_ capture and to valorize the biomass produced. This study utilized the CSMS at bench-scale to develop an experimental approach that may be combined with bioprocess modeling to optimize conversion rates of the algal biomass, and ultimately techno-economic analysis. First, an additional biofilm reactor (BR_cons2_) inoculated with the same photoautotrophic culture was added to the system. In this configuration the biofilms in BR_cons_ and BR_cons2_ each received fresh growth medium, while the gas flowed sequentially through BR_cons_ and Analyzer 2, to BR_cons2_ and Analyzer 3. The addition of Analyzer 3 allows for a direct comparison of the CO_2_ uptake rates in BR_cons_ and BR_cons2_, respectively. As expected, both biofilms consumed CO_2_ at approximately the same rate for the duration of the experiment. The addition of a second photoautotrophic biofilm therefore appeared to double the system’s overall CO_2_-sequestering capacity (Figure 9a). In scenarios with a finite source of CO_2,_ as is the case in this experiment, the CSMS provides a means to determine optimal reactor length or number of modular reactors. This may be relevant to applications aimed at CO_2_ capture downstream from industrial processes where space and cost savings become a factor.

In a subsequent experiment, the system was configured such that both medium and gas passed sequentially from BR_cons_ to BR_cons2_. That is, the medium effluent from BR_cons_ was the medium influent of BR_cons2_. As such, BR_cons_ and BR_cons2_ each represented half of one long biofilm which was approximately 300 cm in length. In this case, CO_2_ and nutrients are finite. Interestingly, when comparing the first half of the biofilm (BR_cons_) to the second half of the biofilm (BR_cons2_), the latter exhibited a diminished rate of CO_2_ uptake (Figure 9b). In this case, more than half of the observed CO_2_ uptake was occurring in the first half of the biofilm. It is possible that some key nutrient(s) had been depleted by the time the growth medium reached the second half of the biofilm, resulting in an overall lower rate of CO_2_ uptake. There is also the potential that the waste effluent from BR_cons_ is in equilibrium with the CO_2_ in the gas phase, causing a subsequent reduction in CO_2_ flux across the silicone membrane in BR_cons2_.

### 3.4. System Utility and Future Considerations

The system described here utilizes a heterotrophic biofilm as a source of concentrated CO_2_. Once this biofilm reaches steady state, its CO_2_ output remains relatively consistent and provides an inexpensive and renewable source of carbon with which to study bio-sequestration by photoautotrophic biofilms. In this sense, the heterotrophic biofilm in BR_prod_ can be viewed as a stand in for any real-world, CO_2_-emitting biological process, such as wastewater treatment or various types of fermentation. When algal systems are installed to capture CO_2_ from flue gas emitted from large industrial point sources, the overarching goal would be to maximize the ratio of CO_2_ captured to CO_2_ released to the atmosphere. While it is possible to increase this ratio by optimizing light exposure (Figure 8a,b) and increasing the biofilm surface area (Figure 9a,b), there is also the potential to re-circulate the gas in a closed loop to achieve near 100% CO_2_ removal.

In algal biodiesel production, lipid accumulation is advantageous and can be induced by starving the algal culture of nitrogen as well as other key nutrients [32]. Cells respond to this stress by directing a higher proportion of their energy and carbon toward neutral lipids, the feedstock for biodiesel production. However, this stress response is also accompanied by a decrease in growth rate, photosynthesis, and CO_2_ fixation [33,34,35]. Hence, a delicate balance must be struck between inducing lipid accumulation through starvation and maintaining a robust biofilm with an adequate growth rate. By depicting a culture’s photosynthetic activity in real time, a system like the CSMS would have significant utility in algal biodiesel production by helping to optimize the balance between starvation, desired product yield through photosynthesis, and collapse of the culture.

In conventional algae culturing, CO_2_ is typically delivered via sparging into the liquid growth medium. This, however, causes a significant amount of CO_2_ to be lost to the atmosphere, a pervasive issue with negative economic and environmental implications [36,37]. This problem could potentially be addressed by mimicking the CSMS’s configuration, where concentrated CO_2_ remains in a closed environment and is delivered to the culture across a permeable silicone membrane. While previous studies have discussed the use of membranes for this purpose [36,38,39], none to our knowledge have applied this specifically in the context of photoautotrophic biofilm CO_2_ sequestration monitoring and optimization. From the data presented, it can be concluded that the system described here is capable of the reliable, real-time detection and quantification of CO_2_ uptake by phototrophic biofilms. This information is critical in understanding how these industrially relevant biofilms respond to changes in their environments (e.g., light intensity), information which is necessary in order to optimize growth and maximize energy- and cost-efficiency. It may also have significant utility in examining the interplay between inorganic and organic carbon utilization in mixotrophic or mixed species phototrophic biofilms, which would be relevant in biofilm systems using a wastewater medium with variable composition.

The fact that the CSMS provides a non-destructive, in situ quantification of CO_2_ uptake makes it a more attractive option than conventional stoichiometric analysis, in which at least a portion of the biofilm must be sacrificed for dry weight measurements [18,19]. The CSMS also allows for a more direct CO_2_ flux analysis than can be obtained from simply monitoring effluent pH, since this is influenced by numerous other factors (e.g., nitrogen assimilation) and in buffered systems pH changes may not adequately reflect CO_2_ dynamics [21]. Some studies have utilized microelectrodes integrated inside the biofilm itself to monitor gas flux and photosynthetic activity [29]. While this approach provides a spatial resolution and resolving power not possible with the CSMS, these electrodes often require frequent calibration and are vulnerable to interference from fouling and gas bubbles [29].

As the effects of climate change continue to increase in severity, there is a critical need for solutions which can slow or prevent the increase in CO_2_ in our atmosphere. Bio-sequestration by photoautotrophic biofilms represents one promising solution which may be a valuable complement to existing CO_2_ management strategies. The biomass generated has inherent commercial value, which can be used to subsidize the process and improve its economic viability [7]. However, process optimization under different environmental and operating conditions remains a major challenge [12]. Technologies like the CO_2_ sequestration monitoring system (CSMS) introduced here can significantly aid in this endeavor and will become increasingly important if we are to effectively mitigate the effects of CO_2_ on climate change.

## Figures and Tables

**Table 1:** CO_2_ sequestration monitoring system (CSMS) properties and operating conditions.

	Inner Silicone Tube	Outer Tygon™ Tube
Colonizable surface area	74 cm^2^	*n*/*a*
Volume	2.90 mL	19.85 mL
Flow rate	0.25 mL/min	2.5 mL/min
Retention time	11.6 min	7.9 min
